# Successful use of point-of-care ultrasound for an elderly patient with heart failure in a primary care setting: a case report

**DOI:** 10.1093/ehjcr/ytae511

**Published:** 2024-09-13

**Authors:** Naoko P Kato, Josefine Svantesson, Peter Johansson, Anna Strömberg, Tiny Jaarsma

**Affiliations:** Division of Nursing Sciences and Reproductive Health, Department of Health, Medicine and Caring Sciences, Linköping University, SE-581 83 Linköping, Sweden; Capio Primary Care Centre Johannelund, Region Östergötland, Skogslyckegatan 5 B, SE-587 26 Linköping, Sweden; Division of Nursing Sciences and Reproductive Health, Department of Health, Medicine and Caring Sciences, Linköping University, SE-581 83 Linköping, Sweden; Division of Nursing Sciences and Reproductive Health, Department of Health, Medicine and Caring Sciences, Linköping University, SE-581 83 Linköping, Sweden; Department of Cardiology, Linköping University Hospital, SE-581 85 Linköping, Sweden; Division of Nursing Sciences and Reproductive Health, Department of Health, Medicine and Caring Sciences, Linköping University, SE-581 83 Linköping, Sweden

**Keywords:** Case report, Handheld ultrasound, Heart failure, Nurse, Primary care

## Abstract

**Background:**

Appropriate assessment of fluid status of patients with heart failure (HF) is challenging in outpatient settings, e.g. primary care, especially among elderly HF patients with multiple comorbidities. The use of handheld ultrasound devices for point-of-care ultrasound (POCUS) has increased.

**Case summary:**

An 80-year-old male had HF with preserved ejection fraction with New York Heart Association (NYHA) classification II. He had multiple comorbidities including chronic obstructive pulmonary disease and been followed up in both a nurse-led HF clinic and a nurse-led chronic obstructive pulmonary disease clinic in primary care. During a scheduled visit to the nurse-led HF clinic in primary care, he exhibited orthopnoea and moderate leg oedema. A HF nurse, using a handheld ultrasound device (Vscan, GE Healthcare), detected B-lines in the left lung, indicating the presence of fluid in the left lung, and an enlarged and non-varying inferior vena cava (IVC) during the POCUS examination. Based on these results, the HF nurse concluded that the patient was experiencing decompensated HF, rather than a chronic obstructive pulmonary disease exacerbation. As a result, his loop diuretics were promptly increased. The patient and his wife received advice on self-care from the HF nurse and the chronic obstructive pulmonary disease nurses. At a follow-up visit 2 weeks later, his breathlessness and swelling were reduced, with no B-lines or dilated IVC found during the POCUS examination.

**Discussion:**

The POCUS can be a good decision support tool for not only physicians but also other healthcare professionals to identify worsening HF and to monitor treatment responses in HF patients in primary care settings.

**Learning points**

- The use of point-of-care ultrasound is helpful for identifying worsening heart failure (HF) and for monitoring treatment responses in patients with HF in primary care settings.
- Point-of-care ultrasound performed by HF nurses can enhance quality of nursing assessment.
- This case emphasizes the importance of individualized self-care support for management of comorbid conditions with a collaboration of specialist nurses in primary care settings.

## Introduction

The number of elderly patients with heart failure (HF) is increasing worldwide. These patients often have multimorbidity such as chronic obstructive pulmonary disease. There is greater emphasis placed on patients with chronic illnesses, such as HF, to be cared for in the community instead of hospital settings.^1^ However, timely and accurate assessment of worsening HF is challenging in primary care.^2,3^ This is partly because of overlapping symptoms among elderly patients with multimorbidity, lack of cardiac specialists, and lack of laboratory equipment in primary care.^3,4^ The use of handheld ultrasound devices for point-of-care ultrasound (POCUS) has increased.^5,6^ The POCUS examinations are predominantly conducted by physicians in hospital-based outpatient clinics. We have earlier demonstrated that HF nurses were able to detect pulmonary congestion and pleural effusion following a brief 4-h training session.^7^ In Sweden, several nurse-led HF clinics have recently been introduced in primary care settings.^8^ This case demonstrates how an HF nurse specialist, within a Swedish primary care setting at a nurse-led HF clinic, effectively manages an elderly HF patient with multiple comorbidities using a handheld ultrasound device.

## Summary figure

**Figure:**
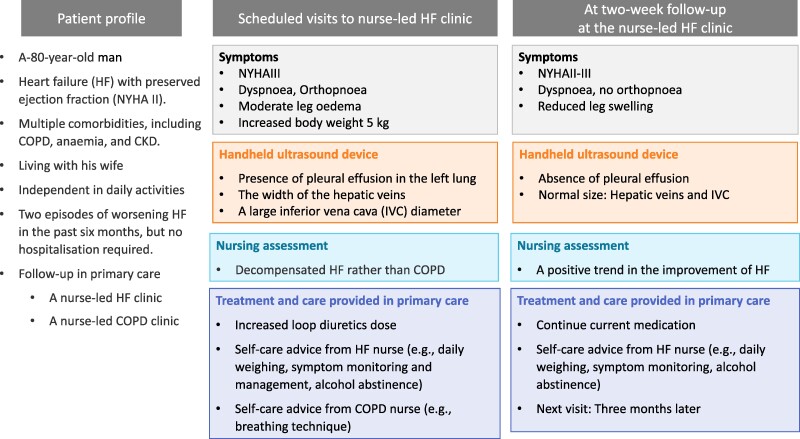


## Case presentation

An 80-year-old male had HF with preserved ejection fraction with New York Heart Association (NYHA) classification II. He received HF medications, including angiotensin-converting enzyme inhibitor and beta-blocker, but not dapagliflozin in 2021, because SGLT-2 inhibitors had not been widely implemented at that time. He had multiple comorbidities, including chronic obstructive pulmonary disease, anaemia, and chronic kidney disease (CKD). He lived with his wife and managed his daily living activities independently. The aetiology of the HF was uncertain, but it was thought to be related to comorbid conditions, such as chronic obstructive pulmonary disease, anaemia, and CKD. Although he had a history of excessive alcohol consumption, he abstained from drinking. His chronic obstructive pulmonary disease was classified as Gold 3, Group E,^9^ based on his forced expiratory volume in the first second (FEV1) of 31% and a history of hospital admission for worsening chronic obstructive pulmonary disease. His forced vital capacity (FVC) was 40%, and the score of the COPD Assessment Test was 17. He used indacaterol and glycopyrronium bromide and, if he experienced signs of worsening chronic obstructive pulmonary disease, would take a beta-2 adrenergic receptor agonist, salbutamol, and beclometasone/formoterol/glycopyrronium bromide (Trimbow®). He had been followed up in both a nurse-led HF clinic and a nurse-led chronic obstructive pulmonary disease clinic in primary care.

Over the past 6 months, the patient encountered two episodes of worsening HF in July and September 2021 without hospitalization. During his scheduled visit to the nurse-led HF clinic in primary care in September 2021, he exhibited symptoms of deteriorating HF. NT-proBNP was elevated to 2671 pg/mL compared to 1426 pg/mL, which was measured when his HF was stable. His HF symptoms had decreased following an increased dose of loop diuretics up to 120 mg/day. The HF nurse specialist and the patient decided a flexible diuretic regimen. In case of a weekly weight gain surpassing 2 kg, the diuretic dosage would be increased from 80 to 120 mg per day. The HF nurse instructed the patient to monitor signs of worsening HF at home and stressed the importance of promptly contacting her in case of deteriorating symptoms.

In early December 2021, the patient visited the nurse-led HF clinic in primary care as planned. He reported shortness of breath since November (see *Table 1*), orthopnoea, and moderate leg swelling. Despite a 5 kg weight gain in the past 2 months, the patient neither increased his diuretic medication nor sought assistance from the HF nurse. He was using salbutamol three times a day at a dosage of 300 μg per day. At the clinic, the patient’s oxygen saturation was 90%, and respiratory rate was 18/min. NYHA had worsened from II to III. The NT-proBNP level was not examined during his visit. For a quick and better understanding of whether these symptoms were due to HF or chronic obstructive pulmonary disease, POCUS was performed by the HF nurse using a handheld ultrasound device (Vscan, GE Healthcare). The HF nurse identified B-lines in the left lung, indicating the presence of fluid in the left lung, along with widened hepatic veins and an enlarged inferior vena cava diameter, indicating increased right atrial pressure. Based on these results, the HF nurse concluded that the patient was experiencing decompensated HF, rather than a chronic obstructive pulmonary disease exacerbation. As a result, his loop diuretics were promptly increased from 80 to 120 mg per day. The patient was advised to weigh himself daily before breakfast. If a 2–3 kg weight gain or increased leg swelling occurred within a couple of days, an extra diuretic dose was recommended. In the absence of improvement within a few days, prompt contact with HF nurses was advised. He was also advised to monitor increased shortness of breath during rest and activity. Considering his history of excessive alcohol consumption, the HF nurse stressed alcohol’s adverse effects on the heart and advised him to maintain abstinence. On the same day, the patient visited the nurse-led chronic obstructive pulmonary disease clinic in primary care, as planned. The chronic obstructive pulmonary disease nurse checked the patient’s breathing technique and recommended monitoring of leg oedema subsequent to salbutamol use, as salbutamol has the potential to exacerbate HF. Both nurses advised him to take medications and inhalations for HF and chronic obstructive pulmonary disease as prescribed. A follow-up appointment was scheduled for 2 weeks later, involving both the HF and chronic obstructive pulmonary disease nurses.

**Table 1:** Case summary

	September 2021	Early December 2021	Mid-December 2021
**Clinical data**			
NYHA classification	II	III	II–III
Body weight	106.5 (+7.5) →97 kg (4 weeks later)	102 kg (+5 kg)	102 kg
Blood pressure		120/60 mg	110/60 mmHg
Pulse rate	83/min	90/min	80/min
Oxygen saturation	92％	90%	93%
NT-proBNP	2671 pg/mL	Not measured	Not measured
**Symptoms**			
Dyspnoea	Yes	Increased	Reduced
Orthopnoea	Yes	Yes	No
Leg oedema	Increased	Moderate	Reduced
**A pocket-sized ultrasound device**			
Pleural effusion		Presence in the left lung	No
Inferior vena cava		Large	Not dilated
Hepatic veins		Dilated	No
**Self-care practices at home**	Had not taken diuretics as prescribed.	Had not increased diuretics. Did not contact HF nurses.	Checked body weight and symptoms every day. Refrained from alcohol use. Good adherence of medication.
**Medication**	Increased loop diuretics up to 120 mg/day. Subsequently, 80 mg/day.	Increased loop diuretics up to 120 mg/day.	Continued to have loop diuretics 120 mg/day.
**Self-care support**			
Advice from HF nurse	Monitor signs of worsening HF. Increase diuretics or contact HF nurses in case of worsening HF.	Check weight daily. Monitor symptoms. Use support stocking, if necessary. Abstain from alcohol consumption.	Continue to daily weight check. Monitor signs of symptoms. Abstain from alcohol consumption.
Advice from COPD nurse		Breathing technique. Use of salbutamol, if necessary, then monitor HF symptoms.	

COPD, chronic obstructive pulmonary disease; HF, heart failure; NT-proBNP, N-terminal pro b-type natriuretic peptide; NYHA, New York Heart Association.

At the 2-week follow-up, the patient presented with reduced symptoms of dyspnoea, coughing, and leg oedema, alongside stable body weight, improved pulse rate, and enhanced oxygen saturation levels (*Table 1*). Lung auscultation revealed no crackles. Point-of-care ultrasound by the HF nurse did not reveal any B-lines or an enlarged inferior vena cava. The patient had measured his body weight and monitored his symptoms every day. Additionally, the patient had abstained from alcohol consumption and taken the prescribed medications as instructed. During the subsequent 6-month follow-up, he did not experience any worsening of HF symptoms, nor were B-lines observed on POCUS.

## Discussion

Managing patients with unstable HF and multiple comorbidities in the community remains challenging, as in this case. Such patients are at high risk of HF exacerbation, underscoring the critical need for accurate and timely physical assessments. Nevertheless, delivering these services in primary care settings is often hindered by lack of resources. Many HF patients, as observed in this case, do not undergo natriuretic peptide tests in primary care settings.^10,11^ As a result, physical examination findings and patients’ reported symptoms play a crucial role in assessing fluid status. Specific physical examinations, such as detecting a third heart sound (S3) and assessing jugular vein distention, may not always be feasible for all nurses. While these assessments are important, they alone do not provide a complete picture. This case suggests that the use of POCUS is helpful for identifying worsening HF and for monitoring treatment responses in patients with HF and chronic obstructive pulmonary disease in primary care settings. In a recent meta-analysis, POCUS has shown to enhance sensitivity of conventional physical examination to assess fluid status in HF patients.^12^ Point-of-care ultrasound might become a good decision support tool, not only for physicians but also for other healthcare professionals in primary care settings.

Few studies have so far reported on POCUS examination by nurses.^7,13^ As in our case, POCUS performed by HF nurses can enhance quality of nursing assessment. The HF nurse specialist had received good ultrasound training and education at university hospital (*Table 2*)^7^; therefore, she could competently use the handheld ultrasound in daily clinical practice at the primary care centre. For the wide implementation of a handheld ultrasound in primary care, educational support is necessary.^14^

**Table 2:** Contents of the 4-h education and training session led by a cardiologist

	Time	Contents
1	Two hours	Understanding the fundamental principles of ultrasound and how to operate the V-scan device. It includes maintenance considerations, adjusting depth settings, switching between cardiac and abdominal modes, and saving recordings.
2	One hour	Understanding and interpreting findings of comet tail artefacts, pleural effusion, and inferior vena cava, along with mastering transducer positioning and handling techniques.
3	One hour	Expert-supervised practice took place with patients admitted to the ward who were experiencing clinically overt decompensation. The nurses independently performed ultrasound examinations. Following this, an expert cardiologist reviewed the nurses’ examinations and engaged in discussions with each nurse about their findings.

This table was created based on the article: Gustafsson M *et al*. Eur J Cardiovasc Nurs. 2015;14(4):294–302.^7^

This case also demonstrates the difficulties for HF patients to monitor their symptoms and take appropriate action at home. The patient had previous experience with deteriorating HF, but both the patient and his wife had difficulty recognizing worsening symptoms at home and did not contact healthcare professionals promptly. This case emphasizes the importance of individualized self-care support for management of comorbid conditions with a collaboration of specialist nurses in primary care settings.^15^

## Lead author biography


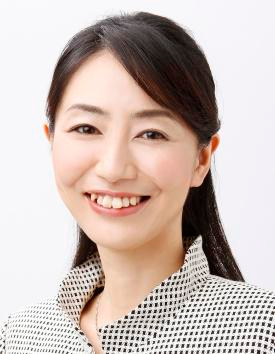


Naoko P. Kato is a nursing scientist and an Associate Professor of Nursing at Linköping University in Sweden. She has a particular interest in using technology to care for individuals living with heart failure.

##  

**Consent:** The authors confirm that written consent for submission and publication of this case report associated text has been obtained from the patient in line with COPE guidelines.

**Funding:** This work was supported by the Promobilia Foundation (to T.J.), the Medical Research Council of Southeast Sweden (FORSS) (753301 to T.J.), the Kamprad Family Foundation (20210053 to N.P.K.), the Swedish Heart Lung Foundation (20210322 to N.P.K.), and the Japan Society for the Promotion of Science (JSPS) Grant-in-Aid for Scientific Research (KAKENHI) (18K17517 to N.P.K.).

## Data Availability

The data underlying this article cannot be shared publicly due to the privacy of the individual who participated in the study. The data will be shared upon reasonable request to the corresponding author.
